# Art Therapy in the Digital World: An Integrative Review of Current Practice and Future Directions

**DOI:** 10.3389/fpsyg.2021.600070

**Published:** 2021-04-08

**Authors:** Ania Zubala, Nicola Kennell, Simon Hackett

**Affiliations:** ^1^Institute of Health Research and Innovation, University of the Highlands and Islands, Inverness, United Kingdom; ^2^Independent Researcher, Moray, United Kingdom; ^3^Population Health Science Institute, Newcastle University, Newcastle upon Tyne, United Kingdom; ^4^Cumbria, Northumberland, Tyne and Wear NHS Foundation Trust, Newcastle upon Tyne, United Kingdom

**Keywords:** art therapy, digital technology, remote delivery, digital arts media, telehealth, online therapy, integrative review

## Abstract

**Background:**

Psychotherapy interventions increasingly utilize digital technologies to improve access to therapy and its acceptability. Opportunities that digital technology potentially creates for art therapy reach beyond increased access to include new possibilities of adaptation and extension of therapy tool box. Given growing interest in practice and research in this area, it is important to investigate how art therapists engage with digital technology or how (and whether) practice might be safely adapted to include new potential modes of delivery and new arts media.

**Methods:**

An integrative review of peer-reviewed literature on the use of digital technology in art therapy was conducted. The methodology used is particularly well suited for early stage exploratory inquiries, allowing for close examination of papers from a variety of methodological paradigms. Only studies that presented empirical outcomes were included in the formal analysis.

**Findings:**

Over 400 records were screened and 12 studies were included in the synthesis, pertaining to both the use of digital technology for remote delivery and as a medium for art making. Included studies, adopting predominantly qualitative and mixed methods, are grouped according to their focus on: art therapists’ views and experiences, online/distance art therapy, and the use of digital arts media. Recurring themes are discussed, including potential benefits and risks of incorporating digital technology in sessions with clients, concerns relating to ethics, resistance toward digital arts media, technological limitations and implications for therapeutic relationship and therapy process. Propositions for best practice and technological innovations that could make some of the challenges redundant are also reviewed. Future directions in research are indicated and cautious openness is recommended in both research and practice.

**Conclusion:**

The review documents growing research illustrating increased use of digital technology by art therapists for both online delivery and digital art making. Potentially immense opportunities that technology brings for art therapy should be considered alongside limitations and challenges of clinical, pragmatic and ethical nature. The review aims to invite conversations and further research to explore ways in which technology could increase relevance and reach of art therapy without compromising clients’ safety and key principles of the profession.

## Introduction

Digital technology is increasingly present in psychotherapy practice worldwide, enabling clients and therapists to connect remotely. This way of improving access to therapy is important for those who might not otherwise be able to benefit from treatment due to living in more remote locations or having disabilities or mobility problems preventing them to attend therapy sessions in person. Despite this general trend of expansion in telehealth provision, to include also psychotherapy services, relatively little is known about its use within art therapy practice (Choe, 2014; Levy et al., 2018). Research in the area focuses primarily on verbal therapies and more specifically on cognitive-behavioral therapy conducted online (Hedman et al., 2012; Saddichha et al., 2014; Vigerland et al., 2016) with some notable examples of work highlighting issues key to psychodynamic psychotherapy (De Bitencourt Machado et al., 2016; Feijó et al., 2018).

Art therapists support clients in engaging in creative processes to improve their psychological wellbeing. Due to incorporating art making within therapy process and the key role of triangular therapeutic relationship between the therapist, the client and the artwork (Schaverien, 2000; Gussak and Rosal, 2016), art therapy practice is arguably more difficult to translate to online situations. However, suggestions have also been made that art therapy is particularly well suited to distance delivery, partially due to increasing ease of sharing images via online channels and non-reliance on verbal communication, and also due to dealing with symbols, metaphors and projections, which can manifest irrespective of medium used (McNiff, 1999; Austin, 2009).

Art therapy profession has not entered the digital world only recently. In fact, it has been critically engaged in often difficult discussions on the risks and potential of digital technology for art therapy practice for over three decades (Weinberg, 1985; Canter, 1987, 1989; Johnson, 1987). Back in 1999 the Art Therapy Journal dedicated a special issue to the links between computer technology and art therapy and has repeated a similar issue a decade later. In 2019, the Journal asked therapists and researchers to consider ways in which professional assumptions can be updated, modernized or reframed to meet contemporary needs.

The use of digital technology in art therapy is not limited to online communication tools but extends to the application of digital media for the purpose of art making, equally relevant to face-to-face practice. While distance art therapy could potentially widen the reach of therapy to include new groups of clients, expanding the range of therapeutic tools to include digital arts media might extend art therapy toolbox to widen access for those clients who might not otherwise engage in traditional art materials for a variety of reasons.

However, it has been argued that the process of digital media adoption in art therapy is slow (Carlton, 2014; Choe, 2014) and resistance to digital technology as well as concerns about the use of digital tools for art making in therapy have been reported in literature (Kuleba, 2008; Klorer, 2009; Potash, 2009). It has been even implied that art therapists themselves may be more conservative and hesitant in their use of digital media than their clients (McNiff, 1999; Peterson et al., 2005; Carlton, 2014). This cautiousness is stipulated to be informed by a heightened sense of responsibility for clients’ safety and wellbeing (Orr, 2016). Art therapists’ own emotional factors and biases were cited to be important barriers to adoption of technology (Asawa, 2009) while it has been suggested that therapists experience “conflict between the desire to promote art therapy and engage in technology and the desire to remain loyal to the field’s origins in traditional methods of communication and art media” (Asawa, 2009, p. 58).

The use of digital arts media is unique to art therapy practice and is perhaps not yet sufficiently researched for that reason, despite its potentially enormous implications for art therapy practice (Kapitan, 2009). Lack of in-depth research on digital art making has been cited as a key barrier for practitioners to introduce digital arts media in therapy sessions (Klorer, 2009; Potash, 2009). Similarly, limited guidelines from professional associations and importance of more specific technology-oriented ethical codes for practitioners are frequently highlighted (Kuleba, 2008; Asawa, 2009; Alders et al., 2011; Evans, 2012).

A challenge identified in early stages of discussion on the use of technology in art therapy was the need for increased collaboration between art therapists, designers and developers in order to device technological solutions suitable to art therapy practice (Gussak and Nyce, 1999). Limited attempts to develop art therapy-specific electronic devices to date lacked in-depth input from art therapists at the technical stage and, in consequence, appropriate integration of the established processes of art therapy with technology (e.g., Mihailidis et al., 2010; Mattson, 2015). In effect, art therapists who incorporate digital arts media in their practice elect to use painting apps not necessarily suitable for art therapy practice. There is also an ongoing debate on the tactile nature of art materials being lost if art is made using digital tools and potential impact on clients (Kuleba, 2008; Garner, 2017). A similar discussion concerns the therapeutic relationship and specifically whether it could be recreated in distance therapy (Klorer, 2009; Potash, 2009).

Despite these indicated debates on the usefulness of digital technology for art therapy practice and polarized opinions, some scholars and practitioners have advocated for increased efforts to incorporate digital art-making in the therapy process suggesting rising and permanent role of technology in art therapy (McNiff, 2000; Kapitan, 2007; Thong, 2007). Given the rapidly growing interest in digital technology applications to art therapy practice, research has been developing relatively slowly and has not yet been systematized. Doing so would help paint an inevitably complex picture of how art therapy is currently engaging with digital technology and how it might make the best use of the opportunities it presents and critically address challenges early in the process.

### Aims

In order to identify key topics important for practitioners and areas for further research, we aimed to capture and synthesize available research literature that explores the role of digital technology in the current and future art therapy practice (understood here as within-session work with clients). More specific research questions were:

- How do art therapists use digital technology in their practice?
- What benefits and challenges of using digital technology with clients do they identify?
- How do clients experience art therapy sessions with digital technology elements?

## Methodology

Through our own experiences in research and practice and following some initial literature searches we were aware that the area we set to explore is complex and relatively novel. Thus, we anticipated that any published research accounts were likely to include a variety of study designs, appropriately to the overall exploratory character of research in the area and in line with research in arts therapies in general, which tends to draw upon diverse methodologies and beyond qualitative and quantitative paradigms, to include also arts-based approaches. We chose an integrative review framework as a guide to allow us to undertake a well-rounded but flexible evidence synthesis that would present a breadth of perspectives and combine methodologies without overvaluing specific hierarchies of evidence (Whittemore and Knafl, 2005). Integrative review is an appropriate method at early stages of systematizing knowledge on a developing subject area (Russell, 2005; Souza et al., 2010) and as such was deemed suitable for our exploratory work which aimed to identify central issues in the area, indicate the state of the scientific evidence across diverse methodological paradigms and identify gaps in current research (Russell, 2005).

### Search Strategy

The following databases were searched for studies published until July 2020: MEDLINE, CINAHL Complete, APA PsycInfo, APA PsycArticles, Academic Search Complete and the Cochrane Library. Google Scholar search, backward and forward reference screening of included publications, and peer consultation were used to identify any other relevant articles. Search string (Table 1) included the four key elements of this review: intervention (art therapy), intervention modification/adaptation (digital technology), methodology (empirical research) and population of interest (all client populations, any setting). These elements of a search strategy were conceptually guided by the PEO (Population-Exposure-Outcome) framework (Khan et al., 2011; Bettany-Saltikov, 2016) instead of the more popular PICO (Population-Intervention-Comparison-Outcome), as the former was considered more suitable for capturing mixed method studies (Methley et al., 2014).

**TABLE 1:** Search string development: concepts shaping this review and corresponding PEO elements.

**Search string**	**Corresponding PEO elements**
TI (‘art therap*’ OR ‘art psychotherap*’ OR ‘arts *therap*’ OR ‘creative *therap*’ OR ‘expressive *therap*’).	E(a): Exposure (intervention): art therapy
AND TI (digital OR online OR technolog* OR remote OR internet OR mobile OR computer OR audio OR virtual OR video OR augmented OR tele* OR *game OR app* OR SMS OR text OR smart OR skype OR distance OR iPad OR tablet).	E(b): Exposure (intervention modification/adaptation): digital technology
AND TX (outcome OR improv* OR increas* OR decreas* OR chang* OR evaluati* OR service* OR intervention OR measur* OR assess* OR effective* OR efficacy OR evidenc* OR impact OR result OR finding OR explor* OR experienc* OR stud* OR pilot OR qualitative OR account OR clinical OR case).	O: Outcomes/methodology: any empirical research
AND TX (health OR ill* OR wellbeing OR well-being OR ‘well-being’ OR mood OR emotion OR ‘quality of life’ OR relationship OR connect* OR social OR esteem OR psych* OR recover* OR mental OR treat*)	P: Population: any client population

*Represents truncation.

### Inclusion/Exclusion Criteria

We opted for broad inclusion criteria to report on all research studies pertaining to the use of digital technology in art therapy and therefore no specific definition of ‘digital’ was adopted other than how authors describe the focus of their paper(s). Time of publication was not initially considered a selection criterion but on reviewing the papers a decision was made to exclude those that focused on technology no longer relevant to modern practice, which, it was felt, related to articles published before 1999.

Articles were included in the review if they:

- concerned the use of modern (currently relevant) digital technology (DT) in within-session art therapy practice with clients;
- reported outcomes observed through empirical study, regardless of whether these were investigated using quantitative, qualitative, mixed or arts-based methods;
- were available online and in English.

Articles were excluded if they:

- focused exclusively on the use of digital technology for office work, assessment, supervision, training or research;
- were PhD theses, dissertations or books/book chapters;
- were theoretical/opinion papers with no empirical data reported.

### Data Extraction

Data were extracted from included papers using a data collection form based on the Template for Intervention Description and Replication (TIDieR; Hoffmann et al., 2014) which helped to record the characteristics of the studies, interventions, outcomes and main findings reported.

### Data Synthesis

We followed the recommended process for synthesizing data in an integrative review (Whittemore and Knafl, 2005) by initially comparing the extracted data item by item, recognizing similarities and groupings, to eventually identifying meaningful categories for studies and interventions included in the review. Each of the papers was read multiple times to generate a mental map of ideas explored across the literature. Iterative process of examining the classified data enabled us to identify themes and relationships which constitute the essence of this synthesis process. Due to expectedly heterogenic character of included studies, attempts at establishing a meaningful classification were at all times guided by the above principles.

## Results

**FIGURE 1:**
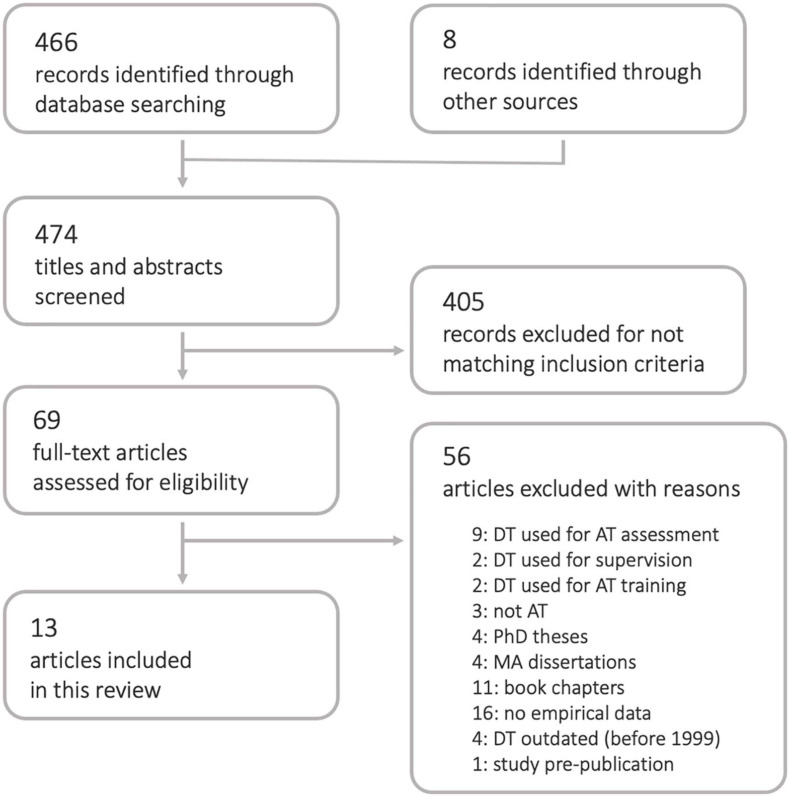
PRISMA flow diagram.

Of 474 records identified through database searching and consulting reference lists, 405 were excluded based on title and abstract screening. Full-texts for the remaining 69 records were consulted and 56 were excluded with reasons (Figure 1). Many of the excluded papers were opinion pieces which did not present empirical outcomes, but were nevertheless helpful in gaining a fuller perspective of the topic and are frequently referred to in the discussion. Selection process resulted in 13 articles included in this review.

### Study Characteristics

All of included research was undertaken either in the US (9 studies) or in Canada (4 studies). The studies were varied methodologically, with qualitative (6 studies), quantitative (1 study) and mixed methods (5 studies) paradigms all represented. The studies employed primarily surveys, focus groups, interviews, case studies and prototyping workshops, often following participatory and mixed-method designs, which seems appropriate for early explorations and for highly applied research with direct implications for clinical practice. Art therapists themselves were research participants in the majority of included papers with only three reporting specifically on client experiences (Darewych et al., 2015; Levy et al., 2018; Spooner et al., 2019). Numbers of participants in qualitative, client-focused and/or workshop-based studies (8 studies) were generally low (ranging from single figures to 25 participants) and numbers of respondents in survey-based studies (4 studies) ranged from 45 to 195. Two papers (Collie and Čubranić, 1999, 2002) reported on the same research study and are referred to jointly throughout this review (including in tables).

**FIGURE 2:**
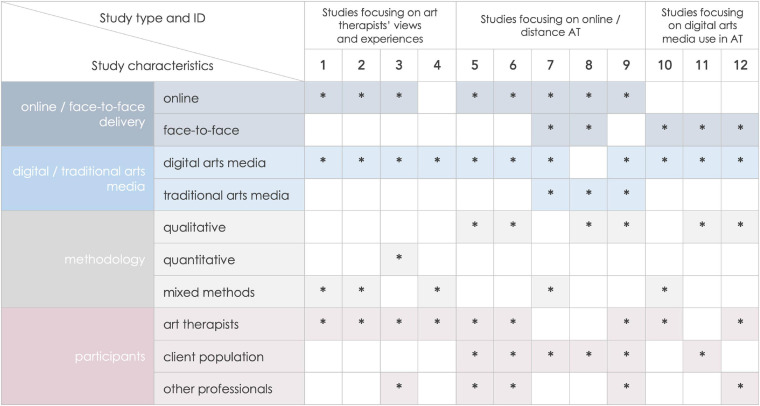
Selected characteristics of included studies: online/face-to-face delivery, digital/traditional arts media, methodology, participant group. *Indicates that a characteristic is present in a study.

The articles tended to discuss the use of digital technology in art therapy practice in a more general way or focus on one of the two uses of digital technology identified in our initial literature review: the use of online tools for distance art therapy and the use of digital media for art making within therapy sessions. Majority of the survey-based studies which examined directly arts therapists’ opinions on the use of digital technology in art therapy were interested in both uses of technology, while workshop-based studies typically focused on either distance delivery or exploration of digital media for art making. There were overlaps and we tried to capture the relationship between the digital technology interest and the categories we eventually decided to group the articles into in Figure 2, which also provides an overview of methodologies and participant groups. The results are presented below in three seemingly separate groups of studies. However, the concepts explored in this research are inevitably intertwined, which is important to note to avoid over-simplifying the nature of opportunities and challenges brought into art therapy realm by the progressing developments in digital technology. Paragraphs below present key messages from the papers grouped in the three categories, except findings pertaining directly to the challenges and benefits of using digital technology within therapy, which will be discussed separately.

### General Views on Technology, Online Art Therapy, and Digital Arts Media

#### Art Therapists’ Views and Opinions

Four articles from two US-based research teams focused entirely on the views and opinions of art therapists on the use of digital technology in art therapy practice and utilized a survey design (Table 2: Peterson et al., 2005; Orr, 2006, 2012; Peterson, 2010). They gathered both the therapists’ experience (based on practice) and expectations (based on personal attitudes). A total number of responses for the four included papers was 474, with majority coming from qualified art therapists and students in art therapy training (in one survey, only 61.5% of respondents were qualified art therapists with the other respondents being not practizing attendees of the AAT conference, Peterson et al., 2005). In one study, follow-up interviews were also undertaken with eight respondents selected according to their readiness for adopting new technologies (Peterson, 2010).

**TABLE 2:** Characteristics of studies focusing on art therapists’ views and experiences.

**Study ID**	**Focus**	**Aim**	**Study design**	**Data analysis**	**Participants**	**Main findings**
(1) Orr, 2006	online AT + digital media	to understand technology as an art media and work tool within the practice of art therapy and to study the need for training in technology	Online survey: questions on current use of technology within art therapy practice and training in technology use	descriptive statistics + thematic analysis for open-ended questions	45 respondents: students and practitioners in art therapy (75% aged 25–45, 92.5% female)	19% respondents used technology as an artmaking tool during sessions, 2.4% used web camera communication during sessions. Respondents were generally using technology within their practices but had very little and insufficient training. Reasons for not using technology were identified, including: cost, limited training, concerns that technology-based artmaking is nonsensory oriented and isolating.
(2) Orr, 2012	online AT + digital media	to determine how art therapists’ perceptions, practices, and training related to the use of digital media in art therapy have evolved	Online survey: questions same as above + additional questions to reflect changes in technology	descriptive statistics + thematic analysis for open-ended questions	98 respondents: students and practitioners in art therapy	Art therapists were increasing their use of digital media in practice with clients, with deeper understanding and questioning of technology (32% used technology as an artmaking tool during sessions, 9.4% used web camera communication, 11.8% used online chat). A range of therapeutic and detrimental aspects of technology in AT with clients were listed. Main barriers: not cost, but lack of training, concerns about ethical and privacy issues and that sensory quality is missing.
(3) Peterson et al., 2005	general use of DT (digital devices and Internet)	to understand the impact of technology on art therapists by exploring how art therapists own and use technology and to determine barriers to ownership and use	Paper survey: questions about personal, professional, and with-client technology use	statistical analyses	195 respondents: 61.5% art therapists, 95% female (survey distributed at AAT conference)	12.3% respondents reported using technology with clients for creating digital artwork and 1.5% reported using web-camera for communication in sessions. Cost and unfamiliarity with digital devices were cited as the most common barrier to device use and ownership. A better understanding of with-client use is needed, how and why digital technologies are adopted and integrated into art therapy practices.
(4) Peterson, 2010	digital media	to determine how art therapists adopt or reject technology and/or new digital media for therapeutic use with their clients	Survey (online+paper) + 8 follow up interviews	statistical analyses; semantic content analysis for interviews	136 respondents, art therapists, of whom 8 took part in telephone interview (3 ‘innovators’, 3 ‘laggards’ and 2 ‘early majority’)	Respondents agreed that if a medium (including digital media) could safely produce a desirable change in a client, then it warranted inclusion in art therapy treatment. Cost was cited as an adoption deterrent, while providing new capabilities for the therapist and the client was an additional adoption factor. Only after therapists feel confident in their personal use of a medium does it become implemented with clients.

Although all studies reported also on the general adoption of technology by art therapists in personal and professional practice including office work, research and training, this review extracted findings pertaining to in-session practice with clients as far as it was possible or to any aspects of digital technology use that directly affect work with clients. Therefore, information on other uses of technology by art therapists, although reported in the cited papers, is not presented here. The general message coming from all included surveys was that art therapists tended to use technology far more often for their own personal practice and for administrative professional tasks than within sessions with clients.

Across the studies, a trend emerged suggesting an increasing use of digital technology within art therapy sessions. A study comparing results from surveys undertaken 7 years apart, found that between 2004 and 2011 art therapists increased their use of digital media in their art therapy practice with clients: from 19 to 32% using technology as an artmaking tool during sessions and from 2.4 to 9.4% using web camera communication during sessions (Orr, 2006, 2012). In addition, in the 2011 survey, 11.8% respondents reported using online chat (Orr, 2012). In an even earlier survey from 2002 (Peterson et al., 2005), 12.3% respondents reported using technology with clients for creating digital artwork and 1.5% reported using web camera for communication in sessions, confirming the rise in in-session technology use over the years.

Two studies highlighted the need for specialist training in digital technology use for art therapists. Orr (2006) reported that in her 2004 survey only 28.5% respondents received some training in using technology to create art, 4.8% respondents felt that the training received met their needs well, while none felt that it met their needs very well. In 2011, the percentage of therapists who reported receiving training in the use of technology as therapeutic tool with clients increased slightly and stood at 36.5% and 11.5% of respondents felt that it has met their needs well (Orr, 2012). Despite this rise in training opportunities, the author concluded that the training “has not kept up with the adoption rate of technology by art therapists” (Orr, 2012, p. 234) and that more and better education is indeed needed.

Another survey conducted almost a decade ago moved beyond establishing how art therapists use digital technology to determine their reasons for adopting or rejecting emerging digital tools for therapeutic use with their clients (Peterson, 2010). A client’s response to a form of digital technology was found to be a key factor in art therapists’ decision as to whether the technology was an effective therapeutic medium. The respondents agreed that if a medium (including digital media) could safely contribute to a desirable change, then its inclusion in treatment is warranted. Cost was, again, cited as an adoption deterrent, while providing new capabilities for the therapist and the client was an additional adoption factor.

A theme consistent across the presented surveys seems to be the highly ethical and professional approach of art therapists in deciding on the use of technology with clients. The responses seemed consistent in indicating that a degree of familiarity with digital medium is necessary for therapists to implement it in therapy session with clients. Importantly, the clients’ response to any novel arts medium is the guiding factor in making decision about a specific technology adoption. Being certain of the benefits for clients seems to be a prerequisite for introducing a specific technology in art therapy sessions. The survey from 2011 revealed that art therapists were increasingly more concerned about ethical and confidentiality issues than 7 years before and that their main reservations about using digital media were linked with uncertainties around ethics (Orr, 2006, 2012).

#### Online Art Therapy: Digital Technology Used for Distance Art Therapy Sessions

We identified five research studies (of which one was reported in two articles) that were concerned primarily with application of digital technology solutions to remote art therapy delivery (Table 3: Collie and Čubranić, 1999, 2002; Collie et al., 2006, 2017; Levy et al., 2018; Spooner et al., 2019). Three of these studies, all from the same Canadian research team, similarly to research discussed above, examined art therapists’ opinions through focus groups (Collie et al., 2006), interviews and participatory designs, including simulated online art therapy interventions (Collie and Čubranić, 1999, 2002; Collie et al., 2017). The studies were concerned with development of an online art therapy service for people with limited mobility, women with breast cancer and, most recently, young adult cancer patients. Two other studies from one US-based research team examined the experience of veterans participating in a blended (primarily online, with face-to-face initial assessment and re-evaluation) creative arts therapies program via semi-structured interviews and a single case study of an art therapy participant (Levy et al., 2018; Spooner et al., 2019). Both studies were undertaken as part of a clinical program evaluation and therefore did not follow a fully experimental design. Although pre-post assessments were undertaken, these have not been reported yet.

**TABLE 3:** Characteristics of studies focusing on online / distance art therapy.

**Study ID**	**Focus**	**Aim**	**Study design**	**Data analysis**	**Intervention**	**Intended population**	**Participants**	**Main findings**
(5) Collie and Čubranić, 1999, 2002	online AT (audio, video) + digital arts media	to design and evaluate a computer system that supports distance group art therapy	Participatory design (PD): simulated two distance art therapy groups (2 h), discussion (1 h) and interviews (30 min)	content analysis	Computer supported distance group art therapy (including audio communication and visual communication in the form of hand-drawn computer images made by the client: “transmitted as they are being drawn (…) rather than as complete images”).	People with limited mobility due to chronic illness, aging, mental health, etc.	10 co-researchers (counselors, art therapists, educators, people with experience of life-threatening illness): 2 groups of 5	Key themes: more freedom and less inhibition when using a computer to make art images; challenges in dealing with silence (”active looking” suggested as solution); qualities of digital images (multiple copies, lack of tactile dimension, etc.); feelings of mastery vs technical problems. Computer supported distance art therapy can include both audio and visual communication and has great potential for people who have mobility issues and those who prefer to have extra privacy. There is a need for suitable ‘social protocols’ (e.g., for looking at other group members’ art).
(6) Collie et al., 2017	online AT (chat only) + digital arts media	to gain outside perspectives on online art therapy methods and to develop online art therapy groups customized for the needs and preferences of young adults with cancer	Participatory design (PD): demonstration/simulation of online AT session (90 min), telephone interviews (30 min), written responses to questions	qualitative thematic analysis	Online AT groups: synchronous, asynchronous, mixed (mix of discussion board, art making and 90 min live chat sessions, based on text-based support groups on CancerChatCanada). Digital art posted by participants on discussion board (either in advance of the live chat session or during the session).	Young adults (18–39) with cancer	7 professionals (recruitment via networks and snowballing): each experienced at least one AT session	Six inter-related themes representing three types of experience (comfort, sense of connectedness and expression) and three types of therapeutic action that supported these experiences (facilitation, group support and dialog about the art). Insights into therapeutic processes in online AT groups, especially with regards to collective meaning-making and sense of connection. Informed further delivery of online AT groups as part of CancerChatCanada (using both digital media and traditional art materials).
(7) Levy et al., 2018	online/blended AT (audio, video) + traditional and digital arts media	to evaluate a creative arts therapy practice as part of improving access to mental health care and rehabilitation for rural veterans	Clinical program with built in evaluation: pre and post assessments (not reported), semi-structured interviews	(not reported)	Individual creative arts therapy (Rural Veterans TeleRehabilitation Initiative Creative Arts Therapy (RVTRI CAT)) via synchronous clinical video telehealth service, 8–10 weekly sessions, and face-to-face re-evaluation.	Veterans (living in rural areas)	20 veterans who conducted at least 50% of their sessions via telehealth (out of 113 veterans in the program)	Key challenges identified and solutions suggested: privacy issues (how to respond to interruptions from family members, attend on time; also - novel layer added to the therapeutic relationship), connectivity issues (offering more than one way to connect, call back if connection lost), image quality lost and therapist not able to observe art making process (share screen, or take photo of art work). Telehealth allows the participant to take a more active role in own treatment process. Shift in triangular relationship between patient, therapist and artwork: patient/artwork relationship is emphasized.
(8) Spooner et al., 2019	online/blended AT + traditional arts media	to illustrate how creative arts therapies practices can be adapted for distance delivery and to demonstrate the potential of this form of delivery	Case studies (1 in art therapy, 1 in dance movement therapy, 1 in music therapy)	(not reported)	Individual creative arts therapy (Rural Veterans TeleRehabilitation Initiative Creative Arts Therapy (RVTRI CAT)) via synchronous clinical video telehealth service (available via smartphones, laptops, tablets), 6–8 weekly sessions.	Veterans (living in rural areas)	1 veteran (1 case study in art therapy)	Distance AT need adaptations to one’s usual process and requires good verbal communication as well as specialist training. It makes care more accessible regardless of barriers such as stigma, distance, disability, and lends itself to community involvement, integration and social engagement. Being able to connect from home allows participants to take a more active role in
								their treatment and to have greater autonomy (Inviting therapist into home environment through telehealth has helped find meaning and rediscover aspects of self that were lost).
(9) Collie et al., 2006	online AT + traditional and digital arts media	to generate both clinical and technological guidelines for distance art-based psychosocial support services for women with breast cancer	Focus groups (3 x 2 h), interviews by e-mail (3 questions) and telephone (30 min–1 h)	systematic inductive approach with content analysis	Synchronous group art therapy via the Internet (with all participants in different places).	Women with breast cancer	25 participants in 3 groups (9 women with breast cancer, 9 art therapists, 5 other therapists, 1 computer expert, 1 graphic designer): age range 31–67, 3 were male	Guidelines for developing distance art-based psychosocial support services for women with breast cancer: allow choice as to means of communication, clearly explain limits to confidentiality imposed by particular communication technology, ensure that participants have access to immediate local support, help participants create suitable private spaces for art making, ensure safety and confidentiality of art that is sent from one place to another, encrypt internet transmissions, art therapists trained in distance facilitation. Other themes: valuing working with physical tactile art materials, accommodate different levels of familiarity with technology, closed groups recommended, opposing views on use of computers in therapy.

In two studies (Collie and Čubranić, 2002; Collie et al., 2017) the participants were also co-researchers, described as art therapists, counselors, educators and people with experience of life-threatening illness (total *n* = 17), who were invited to take part in simulated online art therapy group sessions. The interventions experienced in the two studies were quite different, one being a group art therapy session in which participants communicated and shared digital images created in real time (Collie and Čubranić, 2002), while the other included both synchronous and asynchronous elements, allowing participants to take part in live chat-based session and also upload images to a discussion board outside of scheduled session times (Collie et al., 2017). In both studies participants shared their experience via discussions and follow-up interviews. Another study (Collie et al., 2006) used focus groups and interviews with similarly diverse participants (*n* = 25) to generate clinical and technological guidelines for distance art therapy.

One of the key conclusions coming from the studies was that online group art therapy, being a relatively novel intervention, would require certain adaptations in relation to face-to-face practice (Spooner et al., 2019), for example development of suitable “social protocols” (Collie and Čubranić, 1999), refining of communication procedures (Collie and Čubranić, 2002) and development of “new therapeutic models” (Collie et al., 2006). These adaptations would need to comply with the legal and ethical guidelines, with new telehealth-related guidance eventually required for art therapy profession and initially adapted from related disciplines such as counseling or psychology (Spooner et al., 2019).

Among participating health professionals (including a large proportion of art therapists), there seemed to be quite polarized opinions about the use of computers in therapy, with majority in favor of distance art therapy, but some participants also expressing concerns about “antitherapeutic” character of technology (Collie et al., 2006). Distance delivery was not generally viewed as allowing anonymous participation – in fact, high value was put on close personal interaction regardless of communication technology used (Collie et al., 2006). A sense of connection and “togetherness” was observed in a study of an online group art therapy (Collie et al., 2017), suggesting that the usual therapeutic group factors may be transferable in a distance therapy setup.

In their evaluation of a US-based creative arts therapy program for veterans living in rural areas, Levy et al. (2018) reported primarily positive experiences of using an online art therapy service. Participants appreciated the delivery mode and not having to travel long distances to sessions and described the normally expected positive effects of therapy like increased confidence, improved communication and making sense of emotions through self-expression. A case study of a female veteran participating in the program (Spooner et al., 2019) initially revealed a decrease in perceived quality of life and satisfaction with health, which was attributed by her and her therapist to the actual progress in therapy being made: becoming more aware of emotions and ready to explore more difficult topics to eventually rediscover aspects of herself that were previously lost. These accounts seem to confirm that the therapeutic process can manifest within distance art therapy sessions and therapeutic outcomes can be achieved.

Two papers, published almost two decades apart (Collie and Čubranić, 1999; Levy et al., 2018), proposed that distance art therapy creates subtle shifts within the usual triangular relationship between the client, the therapist and the artwork (Schaverien, 2000). It was suggested that the client/artwork relationship is emphasized, while the client and the therapist are geographically separated and the client remains particularly connected and “co-present” with the art. This could create new opportunities for therapy and mean that the physical separation between the client and the therapist might affect art therapy less than verbal forms of therapy.

#### Digital Arts Media: Digital Technology Used for Making Artwork in Art Therapy Sessions

Three articles focused primarily on the use of digital media within face-to-face therapy settings (Table 4: Choe, 2014; Darewych et al., 2015; Kaimal et al., 2016), but it needs to be noted that the technologies discussed can potentially be successfully applied in distance therapy situations. Two papers examined applicability of iPads and/or other touchscreen devices to art therapy. One study reported on the experiences of adults with developmental disabilities through phenomenological approach (Darewych et al., 2015), while the other set to explore some unique potentially therapeutic features of art applications for iPads from art therapists’ perspective, utilizing the methods of a survey and focus groups (Choe, 2014). The third and most recent study focused on the relevance of virtual reality art-making tools (Kaimal et al., 2016). This small selection of papers nevertheless provides a good overview of the current application of digital media to making art in art therapy sessions and introduces a client perspective.

**TABLE 4:** Characteristics of studies focusing on digital arts media use in art therapy.

**Study ID**	**Focus**	**Aim**	**Study design**	**Data analysis**	**Intended population**	**Participants**	**Main findings**
(10) Choe, 2014	digital media (iPads)	to explore the qualifying features and qualities of digital art materials, specifically art apps on iPads, for art therapy use	Participatory design (PD): questionnaire (on qualities of art apps, client populations most suitable, pros and cons of iPads in AT) and focus groups (4 x 100–140 min)	iterative process: systematic coding, linguistic analysis	(All client groups)	4 responses to survey (arts therapists who have used iPads with clients), 15 participants in 4 focus groups (art therapists and trainees with clinical experience, 14 female)	Advantages / disadvantages of using iPad for AT were identified and client groups that could benefit most. The app’s impact on clients was the most important consideration. Six concrete features of an “ideal” art app for AT emerged: therapist’s control over options; creation of separate, secure portfolio folders; recording of the art process; integration of mixed media and multimedia; assessment capability; privacy and confidentiality.
(11) Darewych et al., 2015	digital media	to explore digital technology as a new art medium and clinical intervention tool in art therapy with adults with developmental disabilities	Phenomenological art-based study: five 1 h individual AT sessions with touchscreen laptops/tablets (free drawing, scribble, mandalas)	in-depth examination of participants direct session comments and artwork	Adults with developmental disabilities	8 adults with developmental disabilities in a community art program: 4 male, 4 female, age 24–49, disabilities: autism (4), Down syndrome (2), not specified (2)	Participants with olfactory and tactile sensitivity favored creating art on texture-free touchscreen devices which offered a compact, mess-free therapeutic environment. Ease of use allowing participants to create images independently was appreciated.
(12) Kaimal et al., 2020	digital media (VR)	to determine the relevance of VR art-making tools to art therapy practice and research, to understand VR from participants’ experiences	Pilot qualitative study: immersive VR art-making sessions using TiltBrush (20–25 min), narrative feedback	thematical analysis	(All client groups)	17 participants: college-educated adults including creative arts therapists, nurses, engineers, physical therapists, administrators and graduate students (age 18–65 years, 5 male, 12 female)	Creating in a virtual environment can induce embodied and novel visual expression, help reduce inhibitions, activate full-body movements, and enhance mood and creative play exploration, not available in the material world. Participants need time to adjust to being in the immersive environment, which can be disorienting, and a proficient facilitator to help them learn the tool and express themselves effectively.

In her investigation on iPads’ applicability to art therapy, Choe (2014) defined three qualities of art apps most valued by art therapists: ease of use or intuitiveness, simplicity, and responsiveness. The therapists who took part in the study believed that it was essential that any art apps were matched with the needs of individual clients and that no single app examined in this project could satisfy the needs of all clients and art therapists. The study found that the therapists had higher expectations of digital than of traditional art materials and were not prepared to compromise on the app’s speed, control or immediacy of working with images. It was suggested that certain client populations may in particular benefit from digital art making in therapy, including, among others, clients with developmental disorders, clients with suppressed immune systems (due to iPads being easier to clean), and clients who have experienced tactile trauma. It was also proposed that digital art making posed risks to some client groups, including those with internet addiction, psychosis or obsessive-compulsive disorder (Choe, 2014). Another study similarly recommended caution about using immersive VR-based tools for art making with clients managing acute psychiatric symptoms (Kaimal et al., 2020).

A study examining the experiences of eight adults with developmental disabilities who used digital art making in art therapy sessions (Darewych et al., 2015), concluded that the participants appreciated the ease of use of the apps tested, which allowed them to create images independently. Those with olfactory and tactile sensitivity preferred the texture-free touchscreen devices to traditional art materials.

Making art in virtual reality, as “a new medium that challenges the traditional laws of the physical world and materials” (Kaimal et al., 2020, p. 17), was also tried and tested for use in art therapy in a small experiential study. The authors propose that therapeutic change can occur in VR environments and that it relates primarily to the unique qualities of the medium and to the fact that the participant is exposed to new environments of choice and creative opportunities not available in the material world (Kaimal et al., 2020).

### Challenges and Opportunities of Using Digital Technology in Art Therapy Practice

The following section presents findings across the three sets of studies that pertain more specifically to the challenges and opportunities of the use of digital technology in art therapy practice. Although these are grouped into three categories, not dissimilar to the categories of studies presented above, findings are based on contributions from across all papers examined in this review. We found frequent overlaps in aspects of technology discussed within papers, for example it was common for studies generally focusing on digital media to provide insights on remote delivery and vice versa. Not wanting to lose those, we decided to thematically analyze the content of all 13 included articles to identify themes relating to the advantages and disadvantages of technology use in art therapy, pertaining in particular to digital media and technologies and processes enabling remote delivery.

#### General Concerns About Including Digital Technology in Art Therapy Practice

##### Cost of equipment

High cost of equipment was cited as the main reason for not including technology in art therapy sessions in a survey from 2004 (Orr, 2006) and from 2002 (Peterson et al., 2005), particularly the cost of electronic art tools advanced enough to allow for true emotional expression (Orr, 2006). However, this issue was not as prominent in a survey from 2011, when it seemed that ethical concerns of art therapists were predominant barriers to introducing technology in therapy sessions (Orr, 2012).

##### Extra time

The importance of a specialist training for art therapists in the use of digital technology is highlighted across studies (Collie et al., 2006; Orr, 2006, 2012; Kaimal et al., 2020). It is recognized that skilful and active facilitation, essential for providing appropriate container (safe environment) and ensuring client safety (Collie et al., 2017; Kaimal et al., 2020), requires extra time for learning (Orr, 2006). Similarly, more effort and time investment in training might be needed on the client’s side, either to adjust to an online mode of therapy (Spooner et al., 2019) or to a new type of digital arts media (Kaimal et al., 2020). A concern has been raised about this additional learning potentially impeding the therapeutic process and that extra time might be needed for establishing a therapeutic relationship (Collie et al., 2006).

##### Technical issues

Unfamiliarity and not being comfortable with the devices were cited as key barriers to engaging technology in art therapy sessions (Peterson et al., 2005; Orr, 2006), which could present a challenge for both the therapist and the client (Spooner et al., 2019). Problems with connectivity, including not having sufficient strength of signal and reliability, were cited as common issues in studies that examined online art therapy (Levy et al., 2018; Spooner et al., 2019). Both inexperience and technical breakdowns could cause distress to clients (Collie et al., 2006, 2017).

#### Concerns Related to Online Art Therapy

##### Confidentiality and safety

Concerns about maintaining confidentiality and privacy in art therapy sessions in which online technology is introduced were raised across the studies (Orr, 2012; Collie et al., 2017; Levy et al., 2018). It was suggested that conducting a session online does not allow for the same assurance of privacy as in a suitable therapy room, due to potential for interruptions from family or housemates (Levy et al., 2018), and that creating a safe emotional container in a cyberspace is harder than in face-to-face therapy (Collie et al., 2017). In addition to confidentiality and safety issues, other ethical concerns have been raised, for example that technology can be used by clients for inappropriate online interactions (Orr, 2012), that the comfort of home environment in case of online sessions might lead clients to behave in ways that they would not in a therapist’s office or that the therapist might potentially observe something concerning or illegal in clients’ private home space (Levy et al., 2018).

##### Technological limitations

A study on online art therapy for veterans highlighted some limitations encountered in how artwork was shared between the client and the therapist, including therapists being unable to view the client’s drawing process as well as their facial expression (Levy et al., 2018). When artworks were made using traditional art media and shown to the webcam, the quality of the image was at times compromised, leading to blur or loss in subtle detail (Levy et al., 2018). Observing art making process directly seemed desirable while not easily achievable in online therapy setting. Levy et al. (2018) also highlighted the importance of the chronological order in which elements are added to the drawing and expressed concern about the therapist not knowing the content of the image until it is completed. In a survey from 2004 a doubt was raised as to whether it would at all be possible for an art therapist to conduct a session without being able to observe art making process in real time (Collie et al., 2006).

#### Benefits of Online Art Therapy

##### Bridging divides/connecting

Research on online art therapy seems to confirm that online mode of delivery has the potential to bridge geographical distances (Collie and Čubranić, 1999; Collie et al., 2017) and expand access to services otherwise unavailable to clients living in rural and more remote areas (Collie and Čubranić, 2002; Levy et al., 2018). It also helps make art therapy more accessible to clients regardless of barriers such as stigma or disability (Spooner et al., 2019), and especially mobility disabilities (Peterson, 2010). It was also observed that technology might have an equalizing effect in a group therapy setting if it is new to everyone (Collie et al., 2017) and that the semi-anonymity of an online group might in fact increase a sense of privacy, particularly for those who are worried about being judged by appearance (Collie and Čubranić, 1999; Collie et al., 2017). Technologies that enable collaborating on a single artwork from different locations or even looking at each other’s art on the screen were reported to bring a sense of connection and emotional closeness, as if being in the same place (Collie et al., 2006, 2017). It was also felt by some that distance delivery promotes community involvement, integration and social engagement by, for example, allowing incorporation of family members into the treatment plan (Levy et al., 2018; Spooner et al., 2019).

##### Therapeutic rapport

Some studies found a positive impact of online mode of art therapy on developing therapeutic rapport (Orr, 2012; Levy et al., 2018; Spooner et al., 2019). The use of technology in therapy was seen by some as comforting and actually helpful in reducing client’s resistance to therapy and/or art making (Orr, 2012). Considering the client’s home environment by the therapist was referred to as an opportunity to establish deeper trust (Levy et al., 2018) and a case study of a female veteran confirmed that her progress was greatly facilitated by the opportunity to invite the art therapist into her home (Spooner et al., 2019).

##### Empowering

Some papers suggested that using technology for distance therapy can be empowering (Orr, 2012), allowing the client to take a more active role in their own treatment process and to have a greater autonomy within and outside therapy sessions (Levy et al., 2018; Spooner et al., 2019). There were also indications that creating art in a home setting might lead to increased engagement in arts processes on a more regular basis and between therapy sessions (Levy et al., 2018; Spooner et al., 2019).

#### Best Practice Recommendations for Online Art Therapy

Two papers in particular (Collie et al., 2006; Levy et al., 2018) attempted to suggest solutions to some of the challenges mentioned above and ways of working which might increase safety and efficacy of online AT practice.

Among the recommendations developed by Collie and her team for distance art groups for women with cancer some seemed potentially applicable to all online art therapy situations (Collie et al., 2006). These included: using a mix of technologies and accommodating clients’ individual preferences, clearly explaining limits to confidentiality imposed by Internet communication, providing guidance to participants for creating suitable private spaces, ensuring that participants have access to immediate local support as an alternative method of addressing emotional safety, and ensuring the safety and confidentiality of art sent from one place to another. The need for training for practitioners in offering art therapy from a distance was also highlighted (Collie et al., 2006). Similar message was repeated in a more recent study, which concluded that the importance of skilful and typically more active than face-to-face facilitation of an online art therapy group calls for specialized training (Collie et al., 2017).

Levy et al. (2018) proposed that in order to address potential technical issues with connectivity, therapists might offer their clients more than one way to connect and agree alternative ways of contact (e.g., by telephone) in case the connection breaks mid-session, to be able to continue any unfinished discussions and/or obtain closure before the end of the session. It was also suggested that interruptions from family could be minimized if the therapist and the client agree in advance how these would be handled, e.g., client could alert therapist when others are present. Instructing clients to be prepared for the session and to call exactly at appointed times was also proposed best practice. To address issues with blurred or unclear image while showing artwork to the webcam, it was recommended that, in case of digital artwork, client might share their screen, and in case of art made with traditional arts media, a digital photograph might be taken and shared with the therapist. Establishing a common vocabulary for describing artwork was another suggestion for improving communication.

#### Concerns Related to Digital Arts Media

##### Lack of tactile qualities

An opinion that technology is cold, isolating, and even “dehumanizing” is repeated particularly in the literature published in the previous decade (Collie et al., 2006; Orr, 2006). These seem to refer primarily to the nonsensory character of digital arts media (Orr, 2006), the lack of tactile and sensual qualities (Collie et al., 2006; Orr, 2012; Choe, 2014) or even lack of tangible physical engagement with the medium as in case of making art in virtual reality (Kaimal et al., 2020). It was suggested that this lack of sensory input might lead to clients disconnecting not only from art materials, but also from their own bodies and social interactions (Orr, 2012) and that the therapeutic value of working with “traditional” tactile art materials should not be underestimated (Collie et al., 2006; Orr, 2006). Technology was also cited as potentially overwhelming and distracting from the creative process (Orr, 2012).

##### Limited room for expression

An observation was made in a paper published over two decades ago that the small size of a computer screen and small mouse movements, used at that time to create images on-screen, could “tame emotions” (Collie and Čubranić, 1999). Similar concern that the standardization of digital tools for art making could impede emotional or creative expression was voiced in forthcoming publications (Collie et al., 2006; Orr, 2012). It was also speculated that a computer image, that exists as multiple copies of itself, might not be an adequate container for emotional material (Collie and Čubranić, 1999) and that using computers for art making might put more emphasis on the product than on the artistic process (Collie et al., 2006). The VR software used for art making was also described as “somewhat crude and clunky” (Kaimal et al., 2020, p. 22), potentially disorienting and incomparable with traditional arts materials in terms of the range of visual effects possible.

#### Benefits of Digital Arts Media

##### Freedom of expression

It was suggested across a number of papers that digital arts media can be empowering by possessing expressive qualities not necessarily achievable with traditional physical art materials (Collie et al., 2006; Orr, 2012). Digital art making, including in virtual reality, was proposed to reduce inhibitions, promote freedom (Collie and Čubranić, 1999; Darewych et al., 2015; Kaimal et al., 2020), and facilitate multimodal expression not limited to images (Collie et al., 2006). It was observed that inhibitions were diminished in creating artwork using digital media since there were no expectations of how a digital artwork should look like and it was also speculated if the elusiveness of a computer image might in fact strengthen the therapeutic process (Collie and Čubranić, 1999). VR environments were found to enhance the freedom of expression without the constraints of the physical world, empower clients with restrictions in their movements and “explore creative opportunities otherwise unavailable in the material world” (Kaimal et al., 2020). Playfulness of the artmaking process and creative exploration was another positive aspect of engaging with digital arts media noted in the literature (Collie and Čubranić, 1999; Kaimal et al., 2020).

##### Digital environment

Some unique technological features of digital environments were cited as presenting key advantages for therapy, including portability, “an all-in-one art studio” (Darewych et al., 2015). Several studies reported therapeutic benefits of a mess-free digital environment for art making, particularly for clients resistant to touching materials (Orr, 2012), those who did not want to get messy during art therapy sessions (Peterson, 2010) and particularly for clients with developmental disabilities combined with tactile or olfactory sensitivities (Darewych et al., 2015). Another potentially therapeutic feature of digital arts media was identified as being able to record and preserve the stages of development of an artwork (Collie et al., 2006), or document work in progress to enhance client’s understanding of how their work has developed over time (Orr, 2012).

## Discussion

This review set out to provide some understanding of how digital technology is applied with therapeutic intent within art therapy sessions. We were able to answer two of our research questions, describing how art therapists work with digital technology in their practice and discussing the benefits and challenges of both online provision and the use of digital arts media. The perspective we were able to provide is the one of art therapists’ primarily and still little is known about clients’ experiences, attitudes and outcomes (Kapitan, 2009; Edmunds, 2012; Carlton, 2014).

Research to date, although some survey-based, is largely qualitative and heterogeneous, presenting difficulties to any inter-studies comparisons. However, these seeming limitations demonstrate, in fact, the seriousness with which the subject has been approached by art therapy practitioners and researchers. Creative use of diverse methodologies to examine art therapists’ views is an essential first step, appropriate for the early stage exploration of how (and indeed, whether) digital technology might be used in art therapy practice. It is appropriate that early investigations are cautious and focused on practitioner’s perspective before any new strategies may be implemented in the actual practice with clients. Such approach seems highly ethical and client-focused, as indeed confirmed in this review in the reasons given by art therapists for their reluctance and cautiousness with which they decide on whether to introduce digital technology in art therapy sessions. Impacts on clients are of primary importance and therapists, understandably, are not willing to compromise on client safety in adopting technological solutions not thoroughly tested (Peterson, 2010; Orr, 2016).

Nevertheless, it is important to highlight that the findings in this review are largely based on art therapists’ opinions and attitudes, not necessarily rooted in experience of using technology in practice. Given the common human error of judgment in terms of imagining theoretical concepts in practice, one can only wonder if some of the opinions expressed might have changed following an actual engagement in digital media-based or online practice, particularly if, as suggested (Asawa, 2009), emotions such as fear and anger might guide art therapist’ initial impressions on technology, and, as suggested elsewhere (Collie et al., 2017), art therapists might be surprised at how quickly they start to feel comfortable with technology that they have had a chance to try out.

As suggested previously, the review confirmed that the perception of digital technology in art therapy realm is dominated by ambivalence and tendencies to pull toward and against, which seems an appropriate attitude on encountering something which we do not yet fully understand. Both an increasing interest in the opportunities that digital technology potentially brings, as well as cautiousness around implementation have been apparent in the literature examined. Nevertheless, a common recognition seems to prevail that, given the likely permanency of digital technology in all aspects of our lives, understanding its benefits and potential harm in therapy situations is indeed essential to reduce risks and increase the therapeutic relevance of digital tools (Kapitan, 2007; Asawa, 2009; Orr, 2012; Kaimal et al., 2016).

In addition to the increased research need, the importance of specialist training for art therapists has been commonly advocated (Orr, 2006, 2012; Kapitan, 2007; Kuleba, 2008; Carlton, 2014; Kaimal et al., 2016). A call has also been made for development of new ethical guidelines for art therapists, which would provide an appropriate framework, aligned with practice needs and with practical considerations (Alders et al., 2011; Evans, 2012). This need for robust guidance, which would help ensure client safety and increase therapists’ confidence in working with technology, has been highlighted more recently by the changing global health situation (COVID-19 pandemic), in which art therapists found themselves transitioning to online practice with unprecedented speed and often against own preference. It is a striking realization that in a survey conducted only 15 years ago none of the respondents reported that they had conducted online art therapy (Peterson, 2006). McNiff’s prediction from over two decades ago that distance art therapy would grow (McNiff, 1999) has, however, become reality, if only too suddenly for some.

This review has synthesized the challenges and benefits of working with clients online, as reported in literature, and any solutions proposed by the authors. It is clear that distance art therapy differs from the usual face-to-face situation on many levels and requires adaptations on both art therapists’ and clients’ side. The relatively novel way of working therapeutically demands more effort and time initially (e.g., for learning of procedures and devices), but has the potential to become less burdensome practically in the long term (e.g., saving the need to travel to sessions). More importantly, it demands skilful and perhaps more active facilitation from art therapists in order to create a safe enough container for clients in virtual space (Collie et al., 2017). It is recognized that this might be harder to achieve in online therapy and compensations might need to be made for the lack of physical presence and limited non-verbal expressions (Chilton et al., 2009). It has been suggested that semi-anonymity that online contact allows might be both restricting and facilitating for the development of therapeutic relationship and emotional connection (Collie et al., 2017; Levy et al., 2018). The responsibility for successful outcomes does not lie entirely with art therapists, and clients might similarly be expected to take on a more active role in their own treatment for a distant art therapy to be beneficial. There is a potential for this increased engagement to promote community integration and to feel empowering for the client (Orr, 2012; Levy et al., 2018; Spooner et al., 2019). The pace of technological advancements also means that certain technical limitations mentioned in the literature may already be overcome, for example observations by some that a computer is not conducive to group therapy (Kuleba, 2008).

As indicated at the beginning of our work, opportunities and limitations of digital technology in art therapy extend beyond telehealth and remote connectivity. The use of digital arts media presents entirely new challenges for the profession and, arguably, entirely new possibilities with potentially profound impacts on practice. There are polarized opinions and ideas around the therapeutic value and risks of incorporating digital arts media in art therapy sessions.

It has been indicated that digital media provide more security to experiment and offer more freedom of expression due to endless modifications and manipulation of artwork being possible, as well as an option to not leave a trace of one’s creative experimentation if one wish (Canter, 1987; Collie and Čubranić, 1999; McLeod, 1999; Parker-Bell, 1999; Peterson et al., 2005; Edmunds, 2012; Orr, 2016). A notion that making digital art may be less intimidating than working with traditional art materials has been widely discussed in literature (Weinberg, 1985; Hartwich and Brandecker, 1997; Collie and Čubranić, 1999; McLeod, 1999; Thong, 2007; Evans, 2012; Orr, 2012; Kaimal et al., 2016). However, it is worth noting that the potentially freeing and playful novelty of digital arts media might not have the same effect nowadays and an observation made in 1999 that people feel less self-conscious due to not having expectations about how a digital image should look like (Collie and Čubranić, 1999) is already likely to be redundant. Similarly, propositions that interaction with digital art making tools gives a sense of mastery and independence (Canter, 1989; Edmunds, 2012; Orr, 2012) might naturally become less relevant with increased use and familiarity.

Nevertheless, the therapeutic potential of making changes to artwork, recording, sharing and revisiting the process of creation, and allowing both the artwork and the process evolve over time, cannot be underestimated (Hartwich and Brandecker, 1997; McLeod, 1999; McNiff, 1999; Evans, 2012; Orr, 2016). Interaction between the person and the electronic device used for art making is potentially therapeutically powerful. It has been suggested that artmaking process becomes a mirror of this relationship (Hartwich and Brandecker, 1997) but also that a computer is simply a mediator in the relationship developing between the client and the therapist (Orr, 2010) or that it can support and provide a transactional space between them (Gussak and Nyce, 1999). The role of the machine in the development of the therapeutic process remains unclear and it will be important to investigate how it affects (or fits within?) the triangular relationship between the client, the therapist and the art.

Probably the most prominent accusation against digital art making tools is their “synthetic” nature, lacking sensual and tactile qualities of traditional arts media, often considered therapeutic in themselves (Kuleba, 2008; Klorer, 2009; Potash, 2009; Carlton, 2014; Orr, 2016; Garner, 2017). Suggestions have been made that this seemingly distant and nontactile nature of digital arts media might result in clients disconnecting not only from sensory experience but also from relationships and the “real world” in the present moment (Klorer, 2009; Potash, 2009). This perception of the isolating, impersonal and even dehumanizing character of digital technology, as well as coldness associated with computers, have been widely discussed by art therapy researchers and practitioners (Gussak and Nyce, 1999; McLeod, 1999; Collie and Čubranić, 2002; Collie et al., 2006; Orr, 2006; Kuleba, 2008). However, some have observed that constant technological advances gradually lead to the cold digital media becoming more integrated with human interactions, senses and emotions, in increasingly intuitive and responsive way (Orr, 2012). Touchscreen sensitivity, for example, allows for pressure to be incorporated in digital art making, mimicking physical art materials, an important quality which was not previously available for art created with a computer mouse, as noted by McNiff two decades ago (McNiff, 1999). Despite some issues which are unlikely to be resolved, it is probably safe to say that with technology generally becoming more human-oriented we may expect an increasing relevance of digital art making tools for art therapy.

An entirely new art medium which is now available within virtual reality environments presents its own unique concerns and prospects (Kaimal et al., 2020), including creative opportunities reaching beyond material world, but also risks of further disconnection from the real tactile experience. Here also some of the previously expresses preconceptions might be challenged, for example another observation made by McNiff that “computer art will never replace the three-dimensional presence of the actual thing being made” (McNiff, 2000, p. 97). It remains debatable of course whether virtual presence is at all comparable to physical experience, but it might be that an opportunity to print out a virtually created artwork using a 3D printer makes the distinction less obvious.

A substantial attention is dedicated in literature to speculation on groups of clients who might benefit most from working with digital arts media. It has been suggested that although this is primarily an individual matter and not necessarily defined by age, contradictory to stereotype (Asawa, 2009), children and young people might be particularly responsive to digital artmaking (Alders et al., 2011; Carlton, 2014). Reports on successful practice with hospitalized children highlight the benefit of adaptations enabled by technology to compensate for physical and emotional challenges (Thong, 2007; Malchiodi and Johnson, 2013). Digital arts media offer a sterile art making environment (Malchiodi and Johnson, 2013; Orr, 2016) and can be used by patients who might not be able to hold art materials but might be able to interact with space or make art on a tablet device using tiny gestures (McNiff, 1999; Hallas and Cleaves, 2017). It has been also demonstrated that the previously mentioned lack of sensory input might be therapeutically beneficial for clients with developmental disabilities and those with olfactory and tactile sensitivities (Darewych et al., 2015). It has been proposed that digital art making tools might be in fact an ideal medium for clients easily overwhelmed by tactile sensations (Alders et al., 2011), allowing them to sustain a safer and longer art making experience (Edmunds, 2012).

Some art therapy practitioners and researchers have long made a proposition that technology-enhanced therapy, whether in form of online delivery or adoption of digital arts media for art making, may actually be the best form of therapy for certain clients and not a mere substitute for more traditional ways of working (Collie and Čubranić, 1999; McNiff, 1999; Parker-Bell, 1999; Evans, 2012). Others have pointed out to contradictory beliefs of some art therapy practitioners, focusing more on potential risks and worrying that technology would “remove what art therapy holds sacred, which is the art.” (Asawa, 2009, p. 64). Between the two polarizing perspectives might be most commonly advocated one, that digital technology is not a replacement for traditional arts media or long established ways of working, but rather an added value, a new quality, expanding and not limiting the profession (McLeod, 1999; McNiff, 1999; Orr, 2006; Choe, 2014).

While flexibility and adaptability have been cited as qualities shared by art therapists that could support them in the predicted continued integration of technology in therapy (Spooner et al., 2019), a question remains whether art therapy profession would accept technology as a true creative and therapeutic medium (McNiff, 1999; Peterson, 2006; Austin, 2009). Over three decades ago, it was suggested that the answer might depend on art therapists’ innate curiosity as artists to investigate the new medium (Canter, 1989) and, more recently, that the potential of technology in art therapy is only limited by practitioners’ creativity and imagination (McLeod, 1999; McNiff, 1999; Peterson, 2010). It has been already proposed that art therapy profession, to remain relevant, might need to “move beyond historically validated media” and also to new contexts (Kapitan, 2007, p. 51).

## Future Research

Given the growing interest in digital technology within art therapy world and the current global health crisis (COVID-19 pandemic) which forced therapists to move their practice online, we expect and would welcome a rise in research in the area. While we already have some understanding of art therapists’ perspective, more research to explore clients’ experiences is clearly needed. This research need must not, however, compromise on clients’ safety and ethical ways of working with technology in art therapy sessions and should observe (and help develop) guidelines from professional associations for the discipline (Zubala and Hackett, 2020). Once new ways of working are established, these need to be reflected in art therapists’ education and research could contribute to identifying the needs for training.

Rise in online art therapy practice could be observed on a large scale in the previous months (second trimester of 2020) and new interventions have been developed with impacts already captured in research which was in press at the time of writing of this review (e.g., Gomez Carlier et al., 2020; Newland et al., 2020). It is important that these accounts of sudden changes in practice are recorded and examined for any lessons to be learned for a longer-term approach to how art therapy might contribute to mitigating the psychological impacts of the pandemic, which are likely yet to emerge (Miller and McDonald, 2020; Titov et al., 2020; Wind et al., 2020; Zubala and Hackett, 2020). The research to follow must acknowledge the extraordinary circumstances under which art therapy has adopted online mode of working, often not by choice but due to demands of the situation and clients’ or employers’ expectations. This fact alone and combined with other factors may have huge implications for practice and we hope that these are captured sensitively in forthcoming research.

Regardless of the mode of delivery, there remains a lot to learn in terms of the emotional and interpersonal implications of digital artmaking for the development of the therapeutic relationship. Previous research encouragingly indicates that therapeutic alliance in verbal psychotherapies can be successfully recreated in an online setting (Sucala et al., 2012). In art therapy case, however, potential impact of technology is not limited to client-therapist relationship but extends to the essence of the triangular relationship including also the artwork. Understanding the impacts of digital tools on the dynamics of this triangular relationship and their place within it seems fundamental to increasing art therapists’ confidence in introducing digital arts media in sessions.

## Limitations

This review attempted to capture research findings from diverse literature for a holistic understanding of the topic (Whittemore and Knafl, 2005) and we recognize that such approach brings some inevitable challenges which we were able to address partially.

Firstly, the heterogeneous character of included studies and breadth of perspectives adopted by the authors meant that the synthesis relied vastly on our own interpretation of the findings due to no specific guidance on such syntheses available. Neither meta-analysis nor meta-synthesis could be performed and instead a method not dissimilar to thematic analysis was employed for identifying key themes often present across the literature examined. It might be that such approach could have missed some of the findings potentially best captured via another methodology. Additionally, inclusion of papers focusing on art therapists’ views and opinions mean that findings are based on both the anticipated and the actual practice-based experiences.

Secondly, we acknowledge that PhD theses, dissertations and book chapters were deliberately excluded from the review due to limited resources and also due to expected further complexities arising from an attempt to synthesize insights from these data sources. The searches have, however, identified substantial volume of material on the subject published in books and available as unpublished doctoral theses and masters dissertations and it would have been valuable to examine these also, perhaps in a more narrative type of review or as part of more specific sub-topic explorations. Similarly, only articles presenting empirical findings were included which means that a number of important opinion papers have not been formally a part of this review. Instead, recognizing the contribution of these authors to the overall conversation, we refer to their work in the extended discussion section. We are also aware that strict inclusion criteria meant that some contemporary uses of digital technology in art therapy such as digital photography, computer animation or digital storytelling, are not discussed here. Peer-reviewed papers in these areas seem sparse despite comprehensive practice-based literature available (e.g., Loewenthal, 2013; Malchiodi, 2018). Therefore, while it was not our intention to exclude these widely used techniques, we acknowledge that this review might not be a complete representation of practice, now commonly adopting many other imaginative uses of digital technology.

Thirdly, we chose not to undertake a formal quality assessment of the studies included, which might have enabled a fairer weight to be allocated to findings, currently considered and presented as being of equal value. An informal quality assessment has been, however, included and we decided that a more formal analysis would not match the complexity of the topic and the nature of the very early exploratory studies, meaning that useful insights might be lost with a standardized form of assessment applied. With progress in research in the area and more methodologically coherent groupings of studies possible, we expect that future syntheses would be able to perform more formalized quality assessments, particularly on studies that report on client experiences.

## Conclusion

This review offers an integrative synthesis of research undertaken to date on the use of digital technology in art therapy, including both online connectivity allowing distance delivery as well as digital artmaking within therapy sessions. The complex characteristics and methodologies of included papers resulted in diverse findings which were integrated to identify key themes in the growing debate on the role of digital technology in art therapy. Potential benefits and challenges were identified, including impacts on the therapy process and the therapeutic relationship. It may be safely concluded that the use of technology in art therapy presents both immense opportunities and serious risks that need to be considered by practitioners, professional associations, and the clients themselves. It is important that early research in the area strives to examine both in order to help art therapists make an informed choice when deciding on whether to incorporate digital technologies in their practice.

We would like to invite the art therapy community worldwide to expand this conversation and to explore together, safely but with curiosity and openness, the expanse of the digital world which, if nothing else, deserves our consideration of its relationship to art therapy. We propose that we approach this exploration with acknowledgment of its importance for the continued relevance of art therapy (Kapitan, 2007) but also reflecting that “art therapy is eclectic and not reducible to a single set of algorithms” (Gussak and Nyce, 1999, p. 194). It might be a demanding but a fascinating journey.

## Author Contributions

AZ conceptualized, planned, and undertook the review, analyzed the data, and wrote the first draft of the manuscript. NK and SH revised the work critically and contributed to edits. All authors contributed to and approved the final version of the manuscript.

## Conflict of Interest

The authors declare that the research was conducted in the absence of any commercial or financial relationships that could be construed as a potential conflict of interest.
